# Helmets in women's lacrosse: what the evidence shows

**DOI:** 10.2217/cnc-2017-0005

**Published:** 2017-04-06

**Authors:** Rebecca L Acabchuk, Blair T Johnson

**Affiliations:** 1 Department of Psychological Sciences, University of Connecticut, Storrs, CT, 06269, USA

**Keywords:** Concussion, helmet, lacrosse, prevention, sport, TBI, women

US Women's lacrosse ranks second only to American football in incidence rate of concussions, according to a study that compiled data from over 200 high schools and 26 colleges [1]. Other studies confirm head injury is a significant risk in the sport; the largest, an epidemiological study using over 1 million athletic exposures in high school and college men's and women's lacrosse over four seasons, found that although women's lacrosse has a no-contact rule, women players had a higher rate of head, face and eye injuries than men; 40% of these injuries were concussions [2]. Stick or ball contact is the primary mechanism of injury to the head in women's lacrosse. In men's lacrosse, a contact sport, most concussions arise from player collisions. Men's lacrosse requires a full protective helmet but, until now, the only approved headgear for women's lacrosse has been eye protection. For the first time this season, women's lacrosse players have the option to wear approved headgear.

Following a concussion, immediate neurological symptoms (i.e., dizziness, confusion, disorientation and blurred vision) generally resolve spontaneously, and no abnormalities are typically found on routine imaging (computed tomography or MRI); however, prolonged symptoms are more likely to occur following a more severe hit or when an athlete has suffered more than one concussion [3]. A growing body of evidence has linked repeated mild traumatic brain injury to debilitating long-term consequences that may silently accumulate; symptoms vary but may include headaches, memory and attention impairment, emotional instability and the progressive neurodegenerative disease chronic traumatic encephalopathy, also known as CTE ([4] and many others). Given the statistics above demonstrating high incident rates of concussion in women's lacrosse, along with the potential for long-term impairment from repeated or severe brain trauma, it is easy to agree on the importance of reducing the risk of head injury in the sport of women's lacrosse. Nonetheless, there is a large debate as to whether adding headgear to the women's game will help solve the problem.

This spring season of 2017 marks the arrival of long awaited new headgear designed specifically for the women's game. The website of US Lacrosse, the sport's governing body, currently states:

“*The ASTM standard is the first ever performance standard for women's lacrosse headgear, developed to help reduce impact forces associated with stick and ball contact in women's lacrosse.*” [5].

ASTM International (originally established under the name American Society for Testing and Materials in 1898) is a global leader in the development and delivery of voluntary safety standards. Women's lacrosse helmets that meet ASTM standard F3137 are now available at sport stores around the country. Although they helped establish the ASTM safety standards for women's headgear in 2015, US Lacrosse has chosen not to mandate its use. This decision leaves parents, players and lacrosse leagues across the country in a precarious position as they are left to weigh the option of incorporating the officially approved headgear into the game. This editorial lists the most common arguments against adopting the use of the headgear in women's lacrosse, examines the scientific evidence relevant to each argument (Table 1) and highlights potential areas of risk under the scenario of ‘optional’ helmet use.

**Table 1.** **Relevant evidence to counter each argument against the use of helmets in women's lacrosse.**

**Argument against helmets**	**Evidence and/or arguments against rationale**
“We don't know the risk of concussions in women's lacrosse”	– Extensive evidence collected from emergency rooms, the High School Rio™ database [6], the Concussion Prevention Initiative [1] and other sports surveillance systems (e.g., 2) show marked risks of concussion in women's lacrosse – Traumatic brain injury and repeated mild traumatic brain injury have been linked to permanent impairments and neurodegenerative disease, including chronic traumatic encephalopathy ([7,8] and many others)

“Helmets do not prevent concussions” Or, similarly: “They wear helmets in men's lacrosse and they still have a high concussion rate”	– The main sources of concussion in women's lacrosse are head or stick strikes [2]. With ASTM standard F3137, the new women's lacrosse headgear was designed and tested specifically to reduce impact to the head by ball and stick, thus reducing biomechanical forces that lead to the most common mechanism of concussion in this particular sport – Goalies are most commonly struck in the head by the ball, yet goalies, protected with superior helmets have the lowest concussion rate of all positions [1] – Helmets will NOT prevent ALL concussions (especially those caused by collisions, which is the main cause of concussion in men's lacrosse), but they may reduce symptom duration or severity of brain trauma

“The best way to prevent concussions is through rules and education”	– Precautions and preventive measures need not be mutually exclusive or singular – Accidental hits to the head by a lacrosse ball can cause significant head injury. Education and proper game technique are important to emphasize, but the inherent risks of a game that involves swinging sticks and launching projectiles necessitates protective gear to prevent unintentional cranial impacts

“The game will become rougher (‘gladiator effect’)”	– The women's helmet will not make players feel invincible because the nose, mouth, jaw, ear and large portions of the temporal and occipital bones remain fully exposed – The risk of increased aggression should be mitigated by consistent enforcement of rules by referees, plus education for officials, coaches and players – Concussion rates are increasing over time due to improved reporting, awareness and diagnosis; this rising trend can lead to the appearance that adding protective equipment increases concussion rate. Such factors must be controlled when evaluating impact of helmets on concussion risk [9]

“The game is about finesse and precision, not physical contact”	– Despite being a noncontact sport, the head injuries have high incidence, even higher than the rate of knee injuries [6]. Rather than player–player contact, the major mechanism of head injury in women's lacrosse is direct impact from a stick or ball. Used correctly and consistently, helmets protect from incidental head impacts that are an inherent risk in the game regardless of physical contact

“Women's lacrosse has not used helmets in the past. We want to honor the tradition of the sport”	– Athletes keep improving (i.e., starting at younger ages, premier leagues, better conditioning) and advanced stick design enables swifter ball tosses; despite their best intentions to play safe, accidents happen. The improving level of athleticism brings with it increased risk of serious head trauma and other injuries

“Players will lose their peers’ respect”	– This claim lacks evidence. Social stigmas and peer pressure impose on discussions of helmet policy, especially when dealing with developmentally vulnerable groups (e.g., middle and high school age girls). All preadult athletes lack adequate frontal lobe development to make proper risk judgments (e.g., to understand the long-term consequences of serious brain injuries)

It is first necessary to clarify some issues, starting with a matter of semantics. Many are referring to the new women's lacrosse helmet as ‘headgear’ rather than calling it a ‘helmet’. This terminology is intended to distinguish the women's ‘helmet’ from the other helmets traditionally used in lacrosse; it is significantly different and less protective than the helmet used by goalies and field players in men's lacrosse. We will use the terms ‘helmet’ and ‘headgear’ interchangeably. The new women's helmet uses a flexible shell in order to straddle two opposing specifications that US Lacrosse set in establishing ASTM safety standard F3137–15; the two competing safety concerns are:

- To develop and test new headgear that meets evidence-based safety specifications aimed to address specific impacts most frequently seen in women's lacrosse;
- To ensure enough flexibility in the headgear to protect unhelmeted players from increased risk of injury (i.e., in the case of head-to-helmet contact).

In attempting to strike a balance to allow only headgear that meets a specified level of protection to the user but limits the risk to those who chose not to wear it, US Lacrosse is able to maintain its current position, which states that headgear use is optional. The introduction of the new helmet design that satisfies the ASTM standard is an advance over the previous uncertified headgear worn in women's lacrosse, but it certainly does not offer full protection (nose, mouth, jaw, ear and large portions of the temporal and occipital bones remain fully exposed). Would a more ideal design to protect against injuries be adopted if all players were required to wear helmets?

As women's lacrosse wades into uncharted territory of mixed use of major protective gear this spring, it is essential to consider whether optional helmet use will produce a new source of injury. Despite the design, helmets worn by some may still harm those who do not wear them. Imagine an entire team of helmet-wearing athletes playing against an entire team without helmets. This scenario appears to be unprecedented in youth, high school and college sports, but can potentially occur under the current rules starting this season. In the case of head-to-helmet contact, the new headgear has a flexible design to help reduce the chance of injuring a player *not* wearing the helmet, but this mechanism of injury should still be of significant concern.

The main argument against the introduction of helmets to women's lacrosse is that no helmet in *any* sport has been proven to prevent concussions. Yet much research stemming from diverse sports helps to establish some generalizations about the biomechanics of head impacts. *While it is true that no helmet guarantees protection against concussion, it is important to consider the biomechanics of head impact that are specific to women's lacrosse.* In full-contact sports such as American football and men's lacrosse, the most common cause of concussion is a collision between players, which often generates high rotational forces upon the head. Rotational force causes brain tissue components, comprised of varying densities (e.g., gray and white matter), to move at different speeds and twist over each other. This ‘shear force’ causes axonal damage to midline internal structures of the brain, especially the corpus callosum [10]. Rotational forces are thought to be the main contributing forces of concussion and are linked to symptom severity [11]. Rotational forces are difficult, if not impossible, to prevent with helmets, which is why there are still so many concussions in sports like American football even though they have the most advanced helmet technology.

In stark contrast, the rules of women's lacrosse prohibit player contact. The primary injury mechanism for concussion in women's lacrosse is caused by an object (ball or stick) striking the head [1]. Head injuries caused by small moving objects such as balls generally involve direct linear force, which eases the job of a proper helmet. Take, for example, batting helmets in baseball or hardhats used by construction workers; both are designed to prevent head injury caused by objects impacting the head. Lacrosse balls are inflexible (like rocks) and almost always fast moving; advances in stick design now enable athletes to fling lacrosse balls at speeds of over 100 miles per hour. Although the rules and equipment vary greatly in men's and women's lacrosse, they use the same balls that can be a significant source of traumatic brain injury unless the player wears a proper helmet.

Concussion statistics offer compelling insight into the protectiveness of helmets. In women's lacrosse, goalie is the position with the lowest concussion rate [1]. Until now, goalies have been the only players in women's lacrosse to wear helmets, and they wear helmets that are, of course, quite different – they are far more protective – than the new headgear that some players will sport this year. As noted, ball and stick blows are the main source of concussion in women's lacrosse; that goalies endure countless driving balls to the head and still have the lowest concussion rate compared with any position on the field provides substantial evidence against the rationale that women's lacrosse should not wear protective headgear simply because helmets cannot prevent all concussions.

The most ardent argument by those against the adoption of headgear is that it will lead to a ‘gladiator effect’, such that women athletes play more aggressively with added protective equipment, morphing what is presently a game of skill and finesse into a game of brute force that could eventually resemble the men's game [12]. But it is important to keep in mind the design of the women's helmet, which in contrast to the men's helmet, is much less likely to lead to feelings of invincibility; as previously mentioned, the nose, mouth, jaw, ear and large portions of the temporal and occipital bones remain fully exposed. The risk is that, if the style of play becomes more aggressive after the introduction of helmets, new sources of injury may well emerge. *The concern of the gladiator effect highlights the importance of education, strict rule enforcement and proper coaching to maintain the integrity and essence of the women's game.* If players on both teams wear helmets, it is easy to imagine referees inadvertently relaxing the rules to allow more aggressive play. Specific education for referees to ensure tight and consistent game calling, along with emphasis on proper coaching and playing techniques, will be essential to mitigate the tendency toward a more aggressive style of play. In order to maintain the spirit of the game with the arrival of the new headgear, consistent rule enforcement is essential whether the headgear is mandatory or optional.

The rising aggression in youth sports in America is not a unique concern to women's lacrosse. As a culture, Americans tend to value winning and compel their children to excel at younger and younger ages. The problem of violence and aggression in youth sports stems mainly from adults [13]. Some youth soccer leagues have instilled ‘silent sideline’ policies to quell disruptive parents. Often, children are rewarded for aggression; parents and coaches laud players who run with reckless abandon, endangering other players. As a culture, America needs to steer the emphasis of youth sports back to skillful play. In short, we need to teach self regulation – when is it appropriate to go hard and when is it necessary to provide space for others. Women's lacrosse has a rich culture of elegant play and finesse. The threat of rising aggression ruining the game – or causing needless injury – is a valid concern, no matter whether or not helmets are used.

Curiously, many opponents of helmet use in women's lacrosse take the argument yet farther, maintaining that strict rule enforcement and education can protect players from injury, rather than mandating the players to wear more protective gear. Precautions and preventive measures need not be mutually exclusive. Despite efforts to increase awareness around concussions in terms of education and rule enforcement, concussion rates in women's lacrosse continue to rise, due in part to increased awareness, better diagnoses and improved reporting [2,9]. Such factors must be considered when evaluating the extent to which helmets affect concussion rates.

It is extremely important to have an accurate indication of youth concussion rates in order to better understand the problem and create targeted safeguards to protect athletes’ from unnecessary injury; the gap in the literature for concussion rates in youth sports is surprising given the rising public health concerns surrounding concussions. The US CDC is requesting funding to create a national concussion surveillance system through the 2017 President's budget [14]. Nonetheless, substantial data currently exist that should be utilized to better inform the current helmet debate in women's lacrosse. Many studies have linked repeated concussions to debilitating long-term consequences such as memory impairment and emotional instability ([7,8] and many others). Animal studies show repeated mild traumatic head injuries cumulate to cause cellular changes in the brain, especially when these happen in rapid succession ([15,16] and many others). Traumatic brain injuries, repeated concussions and high rates of subconcussive hits have been linked to the development of severe impairments later in life, such as chronic traumatic encephalopathy ([7], and many others). Furthermore, the risk for delayed irreversible neurological damage increases with the number and severity of injuries ([17] and many others). With youth sports being played at younger ages and premier sport clubs growing in popularity, expanded seasons have resulted along with more advanced levels of play. Thus, it seems clear that head injury rates will continue to increase in girl's lacrosse as well as other sports. Adding helmets may even attract higher enrollment rates in the sport at the youth level, which encourages the long-term sustainability of women's lacrosse.

Finally, US Lacrosse must consider the strong social pressures that affect both sides of the helmet debate, given that they have not mandated helmet use for women's lacrosse. Young players prioritize fitting in and are too young to comprehend the potential risks. Imagine the negative emotions a young player would feel if she were the only one wearing a helmet, and is told by others that players like her are ruining the game, or she is benched by her coach or ridiculed teammates, all because she ‘chose’ to wear a helmet. Meanwhile, her parents may not let her play unless she wears a helmet, even though the coach is strongly against the use of headgear. It is easy to mock the parents as overprotective, but what if the girl has had a previous concussion and her physician recommended the helmet as a caution?

The sport of women's lacrosse finds itself in a difficult position, but the debate about helmets is not unique to this sport; for years, researchers have recommended helmet use for women's field hockey based on the high rate concussion and potential for serious head injury from unprotected head contact with a high-speed hard ball [18]. What is unique about the present situation in women's lacrosse is: there is now an approved helmet that female lacrosse players of all ages are allowed to wear in practice and in games; the helmet is flexible and protects only part of the head; the helmet was designed and tested to reduce head injury caused specifically by ball and stick impact; and most notably, use of the helmet is ‘optional’.

US Lacrosse helped develop the ASTM safety standard that the new women's helmets on the market now achieve, but US Lacrosse is not mandating or even endorsing the helmet's use. Who, then, will decide if female players will wear the new headgear at the youth, high school and collegiate level, and what factors will drive this critical decision? Should state/federal policy address the issue, such as the state of Florida has done? Will state or regional athletic conferences weigh in on the decision? Should each individual board, local league or sports club make recommendations on helmet usage for youth players? Or will decisions be left up to parents who put their child's safety first, or conversely, staunch supporters of the sporting tradition who ignore or deny relevant evidence? Coaches may be pulled in opposing directions, wanting to protect their players while at the same time maintaining the integrity of the game as they know it. These questions illustrate the unanswered questions and confusion that may ensue in the upcoming season.

In summary, the debate over helmets boils down to a straightforward cost-benefit risk analysis. Why wait until more girls incur head injuries before recommending or mandating the use of safety approved headgear? US Lacrosse should carefully weigh the *existing* scientific evidence to help guide the important decisions being made around the country this spring. In our opinion, the evidence is already more than sufficient to recommend that US Lacrosse should take a firmer stance and mandate helmets, as the benefits outweigh the risks. US Lacrosse should also revise the safety standard to maximize protection without having to satisfy the competing priority of protecting those who choose NOT to wear a helmet. Lacking national leadership on the helmet debate this spring, individual youth leagues must take the lead and make a common decision to adopt the new headgear for the safetly of young children and sake of their sport's future.

## Author's note

The risk of head injury in youth sports is a serious issue. We are seeing changes in protective gear, how rules are structured or enforced, and increased studies informing concussion risk across many sports, not just women's lacrosse. We realize any sport in transition will trigger good and healthy debate about the merits of change and the impact on the players and on the integrity of the sport. Although we all welcome constructive dialog, it is important to base critical safety decisions on scientific evidence, rather than emotion, attachment, and anecdotal evidence. Because the new women's lacrosse headgear is currently only optional, all parties involved in determining its usage (athletes, parents, coaches, board members, policy makers, etc.) must have access to the relevant scientific research to make an informed decision. Our aim in writing this article is to inform this discussion; we provide a brief overview of the background on this topic and compile relevant details from the scientific literature, and thus avoid using purely anecdotal or emotional arguments. We reviewed the evidence from both sides of the debate and have done our utmost to present scientific findings in an unbiased manner.
